# Single and combined effects of metal-based fungicides on *Eisenia andrei* in different scenarios of climatic change

**DOI:** 10.1007/s11356-024-35309-z

**Published:** 2024-10-16

**Authors:** Hussain Kaka, Prosper Opute, Mark Maboeta

**Affiliations:** 1 Unit for Environmental Sciences and Management, North-West University, Potchefstroom, 2520 South Africa; 2 Department of Animal and Environmental Biology, Faculty of Life Sciences, University of Benin, Benin City, Nigeria

**Keywords:** *Biomass*, *Eisenia andrei*, Reproduction, Growth, Avoidance behaviour, Climate change; copper oxychloride; mancozeb

## Abstract

This study evaluated the ecotoxicity of metal-based fungicides under the current scenarios of global climatic change (20 °C and 25 °C) and moisture content (30% and 50%) in single and binary mixtures of copper oxychloride (CuOx) [200, 500 and 1000 mg/kg] and mancozeb (MnZn) [44, 850 and 1250 mg/kg]. Endpoints assessed included mortality, changes in biomass, avoidance behaviour, and reproduction utilising standardised protocols (ISO and OECD). The changes in biomass and mortality tests lasted 28 days, followed by a 28-day reproduction test and a two-day avoidance test. In all temperature-moisture combinations, the mortality rate in the exposure groups exceeded 10% only in the CuOx1000 and CuOx1000 + MnZn1250 mg/kg groups. However, at 20 °C and 30% moisture, the mortality rate exceeded 10% only in the CuOx500 + MnZn850 mg/kg treatment. Relative growth rates in the CuOx and MnZn treatment groups decreased with increasing concentrations. In CuOx MnZn and the binary mixture treatments at 20 °C 30% and 25 °C 50% conditions, avoidance response behaviour was greater than 80% throughout the exposure, except in CuOx200 mg/kg, MnZn44 mg/kg and CuOx200 + MnZn44 mg/kg. The reproduction of exposed earthworms in all treatment groups was concentration-dependent and influenced by varying temperatures and soil moisture conditions. No juveniles or cocoons were produced in the CuOx1000 mg/kg treatment at 25 °C 30%, indicating that copper oxychloride may be more toxic than mancozeb, especially in drought conditions. This study found that different temperatures and soil moisture levels altered the ecotoxicity of CuOx and MnZn. It can be concluded that climate change is likely to significantly impact the outcomes of metals to earthworms and their ecological activities.

## Introduction

Modern agricultural practices in recent decades have been inextricably linked to a wide range of synthetic pesticides. Although there are increased efforts at regulation in many regions, their application and effects on soil and soil fauna remain unabated due to the increasing demand for food to cater for the ever-growing world population. Continuous exposure of nontarget soil fauna to pesticides is considered a disadvantage of recent agricultural advances. However, the connection between increased crop yield benefits and pesticide use drawbacks has remained contentious (Morgado et al. 2018). Soils support a wide variety of biological life and provide a variety of ecosystem functions and services that enable terrestrial life to thrive, playing significant roles in supporting primary productivity and modulation of nutrient flows (Opute et al. 2022). Therefore, accumulating chemical contaminants such as metal-based fungicides in soils can adversely affect key soil organisms such as earthworms and microorganisms, thus affecting overall ecosystem health.

Mancozeb (MnZn) is a member of the ethylene-bis-dithiocarbamate (EBDC) class of pesticides belonging to the subclass of dithiocarbamate fungicides with a wide range of applications. It is a broad-spectrum compound comprising 20% manganese and 2–5% zinc salt. Mancozeb is widely used to control fungal diseases in agricultural, ornamental, and horticultural crops (Mohammadi-Sardoo et al. 2018). Because mancozeb is a potent inducer of oxidative stress, there is an urgent need to develop highly sensitive biomarkers for early detection and biomonitoring in the environment (Mohammadi-Sardoo et al. 2018). Copper oxychloride is another widely applied metal-based fungicide widely applied in agriculture, especially in vineyards, to prevent and treat downy mildew (Proffit et al. 2015). The widespread use of copper oxychloride can result in an increase in copper accumulation in soil, which can have a negative impact on the soil mesofauna (Oladipo et al 2019). Since the use of copper-based fungicides is still allowed in organic farming in most countries, a reduction in copper accumulation in agricultural soils is unlikely (Wang et al. 2018). The exposure of these pesticides to nontarget soil biota remains a critical concern. However, the exposure to mixtures of pesticides in modern agricultural regimes is of utmost importance. This is because their effects do not always reflect the toxicity of their constituents but rather the antagonistic or synergistic effects of the mixture, which may result in unexpected consequences. Current agricultural practises are highly optimised, with a heavy reliance on the use of a variety of agrochemical products, including fungicides. However, despite the reported effects of mixture toxicity, the complexity and specific characteristics of the interaction of these chemical compounds remain a significant challenge for ecotoxicologists and risk assessors (Morgado et al. 2018).

Earthworms are excellent bioindicators for determining the toxicity of mixtures of multiple contaminants (González-Alcaraz and Van Gestel 2016). The international standardisation guidelines recommended *Eisenia andrei* for laboratory ecotoxicity assays (ISO 2012; OECD 2016). *Eisenia andrei* is recommended due to its rapid growth and reproduction, adaptability to laboratory conditions, and sensitivity to various soil contaminants (Lisbôa et al. 2021). Many studies have used *E. andrei* to assess the impact of various environmental factors on pesticide toxicity (Hennig et al. 2022). *Eisenia andrei* have been used for the evaluation of the ecotoxicological risk of copper oxychloride and mancozeb pollution in soils (Reinecke et al. 2002; Maboeta et al 2004; García-Santos and Keller-Forrer 2011; Wang et al. 2012; Berenstein et al. 2017; Carniel et al*.* 2019; Oladipo et al 2019). The previously referenced studies were carried out under standard laboratory conditions based on the OECD 222 guidelines (Reinecke et al. 2002; Maboeta et al. 2004; García-Santos and Keller-Forrer 2011; Wang et al. 2012; Berenstein et al. 2017; Oladipo et al. 2019). However, climate change could alter the effects of these tested pesticides on earthworms (González-Alcaraz and Van Gestel 2016). The negative impacts of various contaminants can increase due to the predicted changes in temperature and soil moisture content (Hackenberger et al. 2018). The present study investigated how changes in air temperature and soil moisture conditions affect the ecotoxicity of mancozeb and copper oxychloride fungicide spiked soils on *E. andrei.* This is based on the exposure concentrations from previous studies (Maboeta et al. 2004; Oladipo et al. 2019) under different climate change scenarios as predicted by the Intergovernmental Panel on Climate Change (IPCC) (Niang et al. 2014), thus placing soil toxicity in a context of near-real-time global change. Previous studies (De Silva et al. 2009; González-Alcaraz and van Gestel 2015; Velki and Ečimović, 2015; González-Alcaraz and Van Gestel 2016; González-Alcaraz et al. 2018; Hackenberger et al. 2018; Bandeira et al. 2020) have tested climate change variables (temperature and moisture). They tested 20 °C, 25 °C, 30% and 50% soil moisture. The tests of this study were carried out for the single and combined pesticide mixtures under four different climate change scenarios: (1) 20 °C + 50% WHC; (2) 20 °C + 30% WHC; (3) 25 °C + 50% WHC and (4) 25 °C + 30% WHC. Earthworm mortality. The influence of temperature and soil moisture on the toxicity of mancozeb and copper oxychloride to earthworms was evaluated, focusing on biological endpoints such as mortality, changes in biomass, reproduction, and avoidance behaviour.

## Materials and methods

### Soil preparation

All exposures in this study were prepared using artificial soil according to the Standard Guideline of the Organisation for Economic Cooperation and Development (OECD 222) (OECD 2016). The artificial soil was prepared seven days before the start of the experiment and consisted of three substances (based on the dry weight): 10% sphagnum peat, 70% silica sand, and 20% kaolin clay. These three substances were thoroughly mixed to ensure a uniform substrate. The pH of the soil was measured (Hanna instruments edge pH meter). It was measured in a mixed sample in a 1: 5 ratio using 1 M solution potassium chloride (KCl) or 0.1 M calcium chloride (CaCl_2_) (OECD 2016). The pH of the soil was between 6–6.5; if not, it was adjusted using 0.3–1% pulverised calcium carbonate (CaCO_3_) (OECD 2016). Water holding capacity (WHC) was also done using the (OECD 2016) guidelines. To determine the WHC, a moisture analyser was used with triplicate samples of 5 g of each concentration. Filter paper-sealed tubes were immersed in water for three hours, then placed in moist silica sand for two hours to reach 100% WHC. Moisture content was measured using a Sartorius MA35M-000230V2 analyser. Based on these results, 30%, 50%, and 60% moisture content levels were calculated.

### Spiking of soil samples

In this experiment, two fungicides were used, viz. copper oxychloride and mancozeb. These are commercial products used as fungicides. The commercial names of these fungicides were Villacrop Villa Unizeb 750 WDG (mancozeb [dithiocarbamate] 750 g/kg) and Villacrop Copper Oxychloride 850 WP (copper oxychloride 850 g/kg). Copper oxychloride was used at concentrations of 200 mg/kg, 500 mg/kg, and 1000 mg/kg, while mancozeb was used at concentrations of 44 mg/kg, 850 mg/kg and 1250 mg/kg. Concentrations were based on previous studies (Helling et al. 2000; Vermeulen et al. 2001; Maboeta et al. 2003; Eijsackers et al. 2005; Oladipo et al. 2019) and the conditions adjusted to simulate varying temperature and moisture scenarios, mimicking the potential impacts of climate change on fungicide toxicity. Copper oxychloride (CuOx) and mancozeb (MnZn) were prepared in single and binary mixtures (CuOx200 + MnZn44, CuOx500 + MnZn850 mg/kg and CuOx1000 + MnZn1250 mg/kg). These single concentrations and mixtures were dissolved in distilled water and added to soil at 30% and 50% moisture content. Control tests were conducted with artificial soil and no added fungicides.

### Earthworm bioassay

The earthworm growth test was carried out using OECD guideline 222 (OECD 2016), and the avoidance test was carried out following the ISO guideline (ISO 527–2, 2003). Adult earthworms (*Eisenia andrei*) with a visible clitellum were used in these experiments. Earthworms were purchased from Ecolife Vermikompos Pty Ltd, Boskop, South Africa. Three replicates per concentration were tested. Ten earthworms were placed in each container and individually preweighed to ensure that the weights were between 0.250–0.600 g (OECD 2016).

1-L containers with perforated lids allowed oxygen exchange for earthworm respiration. The lids were closed to prevent earthworm escape. The tests were carried out at 20 °C and 25 °C in climate-controlled rooms. The soil moisture was monitored at 30% or 50% for the entire experiment, and deionised water was added using a spray bottle on the soil's surface if necessary.

The earthworm bioassay lasted 28 days. Earthworms were fed 5 g of horse manure on day one and then every seven days until the end of the growth test. After 28 days, the adult earthworms were removed, and the cocoons and juveniles in the containers were counted by hand-sorting and placed back in the container. Five grams of manure (as feed for emerging juveniles) was added to each container and left for another 28 days. No further feeding was performed for the remaining duration of the reproduction test. To calculate the average number of juveniles per cocoon, the total number of juveniles produced was divided by the total number of cocoons found at the end of the reproduction test.

The avoidance test followed ISO (2012) guidelines. Adult clitellate earthworms were exposed to the same concentrations as in the growth experiment. A two-chamber container (1L, 0.02 m^2^ cross-sectional area) was used with a 50–60 mm soil depth. A plastic divider separated the control and test soils (250 g each). After adding soil, the divider was removed, and ten earthworms were placed in the middle. Containers were incubated for 48 h at the desired temperature. Afterwards, the divider was replaced, and earthworms in each half were counted. Earthworms found in the middle were counted as half for each side. Missing earthworms were recorded as escaped. The avoidance response was calculated as follows (Eq. 1):

1 $$AR=\frac{{N}_{c}-{N}_{t}}{N}X 100$$

*AR*: Avoidance Response
*N*_*c*_: Number of earthworms in control soil
*N*_*t*_: Number of earthworms in test soil
*N*: Total number of earthworms (control soil + test soil)

For the growth test, ten adult earthworms were added to each container (500 ml) and filled with 500 g of artificial soil. The earthworms were fed with horse manure. The manure was air-dried before being used. Each container received 5 g of finely crushed and moistened horse manure every seven days for the 28-day growth test. This seven-day feeding cycle was not necessary for the avoidance or reproduction tests. The manure was moistened with distilled water. Containers were observed every week (days 7, 14, 21 and 28), and any uneaten food was removed from the container. The containers with uneaten feed had the ratio of feed reduced to avoid fungal growth. The relative growth rate (RGR) of the earthworms was calculated as follows (Eq. 2):

2 $$RGR=\frac{(Wt-Wo)}{Wo}X 100{\%}$$

RGR: Relative growth rate
Wt; Initial average weight of earthworms (day 1)
Wo: the average weight on days 7, 14, 21 and 28

The earthworms were removed from their containers every seven days, cleaned with distilled water, and dried on paper towels. They were weighed before returning them to their respective containers; however, any earthworms that did not respond after anterior poking were recorded as dead. After the 28-day period, the mature earthworms were removed from the container, and only juveniles and cocoons were left behind. The cocoons were left for another 28 days before the juveniles were counted and recorded for reproductive success.

### Statistical analysis

Statistical analyses for the earthworm assays were performed using the SigmaStat® programme Version 4.0 (2016) (Systat Software Inc., CA, USA). To compare the different groups, the program assessed data normality. Based on this assessment, either a one-way analysis of variance (ANOVA) or the Kruskal–Wallis test was performed. Average weight and standard deviation (SD) were calculated for all the data. The relative growth rate (RGR) was used to assess biomass changes.

## Results

### Bioassays

#### Mortality

**Fig. 1:**
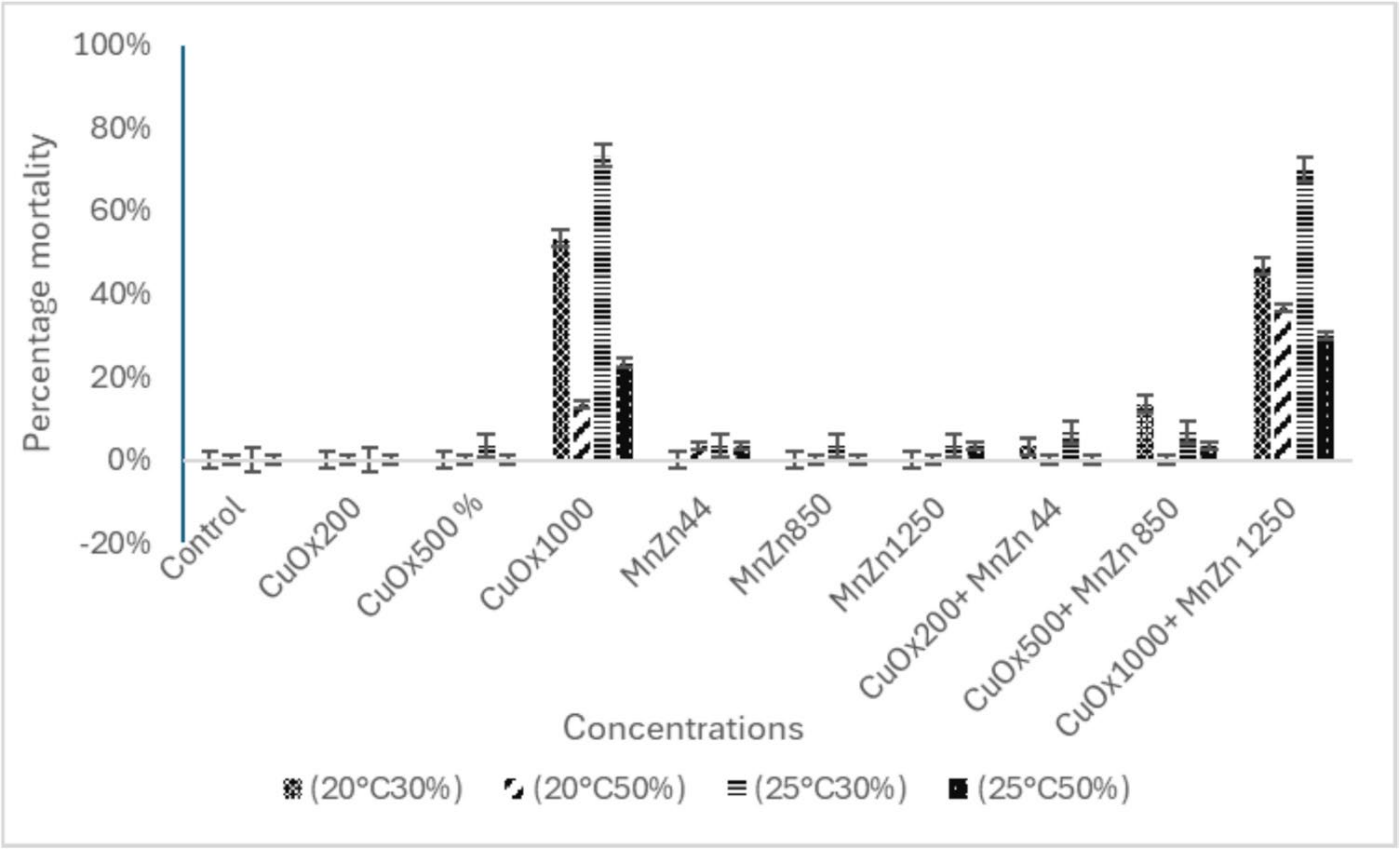
The percentage mortality of *E. andrei* to copper oxychloride (CuOx) and mancozeb (MnZn) in single and binary mixtures under different temperatures (T) and soil moisture (SM) conditions over a 28-day period. Different colours indicate the different treatments (20 °C 30%, 20 °C 50%, 25 °C 30%, 25 °C 50%) (*n* = 30)

The percentage mortality of earthworms exposed to copper oxychloride and mancozeb in single and binary mixtures over a 28-day period at various temperatures and soil moistures is reported in Fig. 1. According to the OECD 222 guidelines for the validity of the test (OECD 2016), mortality should not exceed 10% in the control. This was achieved in this study and is indicated in Fig. 1. The controls did not have any fungicides added and were thus only artificial soil. Therefore, this value as an endpoint was considered a significant response. Mortality in the control, CuOx200, CuOx500, MnZn44, MnZn850, MnZn1250, CuOx200 + MnZn44 and CuOx500 + MnZn850 mg/kg groups was lower than 10% in the different combinations of temperature and moisture. Based on the results, CuOx was responsible for most of the recorded earthworm mortality in all treatment groups. However, the highest significant mortality was observed in the mixture treatment, CuOx1000 + MnZn1250. The combination of copper oxychloride and mancozeb did not show increased toxicity when tested under different climate conditions. However, a detrimental interaction between the two fungicides was observed when the moisture level was reduced to 30%.

#### Biomass changes of earthworms

The changes in biomass of earthworms after the acclimatisation period indicate that there were no significant differences (*p* > 0.05). The difference between the sample groups was determined with a one-way analysis of variance (ANOVA) between the controls used in this study (20 °C 30%; 20°50%; 25 °C 30%; 25°50%). Therefore, the control results in Tables 1-3 were considered valid. Table 1 summarises the relative growth rate of CuOx treatments under different temperature and soil moisture conditions. CuOx200 mg/kg, CuOx500 mg/kg, and CuOx1000 mg/kg under 20 °C 30% were significantly different (*p* < 0.05) from each other and the control after 28 days of exposure duration. At increased moisture conditions (20 °C 50%), only the CuOx1000 mg/kg treatment was significantly different (*p* < 0.05) from the control group. However, under increased temperature/reduced moisture conditions (25 °C 30%), the CuOx500 and CuOx1000 mg/kg treatments were significantly different (*p* < 0.05) from each other and the control sample. At increased exposure to temperature/moisture (25 °C 50%), there was a significant difference (*p* < 0.05) between CuOx200 and CuOx1000 mg/kg and the control. By comparing the three treatments (CuOx200 mg/kg; CuOx500 mg/kg; CuOx1000 mg/kg), there was a significant difference (*p* < 0.05) between 20 °C 30% and 20 °C 50%, and between 25 °C 30% and 25 °C 50%.

**Table 1:** The relative growth rate (RGR) (%) of E. andrei in copper oxychloride (CuOx) treatments (CuOx200 mg/kg; CuOx500 mg/kg; CuOx1000 mg/kg) over a 28-day period under different temperatures and soil moisture conditions. (*n* = 30)

	RGR (%)				
Treatment (mg/kg)	Day 1	Day 7	Day 14	Day 21	Day 28
20 °C 30%					
Control	0.46	-3.78	-8.27	-19.41	-28.93^a■^
CuOx200	0.43	-5.88	-11.89	-20.59	-28.55^b●^
CuOx500	0.44	-19.34	-26.74	-32.06	-40.75^c▲^
CuOx1000	0.40	-31.06	-46.35	-52.93	-59.07^d♦^
20 °C 50%					
Control	0.38	5.77	0.53	-10.70	-20.06^a■^
CuOx200	0.40	-4.03	-9.67	-15.74	-25.09^a■^
CuOx500	0.41	-10.46	-17.01	-24.37	-31.73^a■^
CuOx1000	0.38	-22.31	-28.12	-34.58	-41.50^b●^
25 °C 30%					
Control	0.30	23.06	16.55	10.62	1.74^a■^
CuOx200	0.32	5.99	-0.36	-7.09	-14.32^a■^
CuOx500	0.31	1.90	-3.80	-14.30	-24.37^b●^
CuOx1000	0.31	-20.60	-29.57	-30.48	-34.29^c▲^
25 °C 50%					
Control	0.27	23.49	12.93	4.51	15.80^a■^
CuOx200	0.27	16.84	12.04	4.49	-1.56^b●^
CuOx500	0.46	-16.84	-23.90	-25.78	-35.91^a■^
CuOx1000	0.47	-22.19	-31.49	-33.74	-40.00^b●^

Different superscript letters^a−d^ (rows) and symbols^■●▲♦^ (columns) (Day 1 – Day 28) indicate significantly different values (*p* < 0.05). Superscript letters a-d (rows) and symbol (columns) (Day 1 – Day 28) show significant differences (*p* < 0.05)

Table 2 summarises the relative growth rate of earthworms in the MnZn treatments under different temperatures and soil moisture scenarios. At 20°C30% and 20 °C 50%, only the MnZn1250 mg/kg treatment group showed a significant difference (*p* < 0.05) with the controls after 28 days of exposure. At increased temperature/reduced moisture (25 °C 30%), all three treatments (MnZn44 mg/kg, MnZn850 mg/kg and MnZn1250 mg/kg) showed significant differences (*p* < 0.05) from the control. However, there was no significant difference between treatments and the control under increased temperature/moisture conditions (25 °C 50%).

**Table 2:** The relative growth rate (RGR) (%) of *E. andrei* in the mancozeb (MnZn) treatment (MnZn44 mg/kg; MnZn850 mg/kg; MnZn1250 mg/kg) over a 28-day period at different temperatures and soil moistures. (*n* = 30)

	RGR (%)				
Treatment (mg/kg)	Day 1	Day 7	Day 14	Day 21	Day 28
20 °C 30%					
Control	0.46	-3.78	-8.27	-19.41	-28.93^a■^
MnZn44	0.40	4.33	-1.17	-9.61	-24.53^a■^
MnZn850	0.46	-16.32	-19.66	-25.46	-30.11^a■^
MnZn1250	0.39	-15.09	-21.20	-28.39	-35.58^b●^
20 °C 50%					
Control	0.38	5.77	0.53	-10.70	-20.06^a■^
MnZn44	0.38	10.58	1.68	-12.91	-21.94^a■^
MnZn850	0.39	-9.42	-14.52	-21.63	-27.86^a■^
MnZn1250	0.38	-10.43	-17.36	-24.34	-32.57^b●^
25 °C 30%					
Control	0.30	23.06	16.55	10.62	1.74^a■^
MnZn44	0.30	12.36	1.87	-2.09	-8.83^b●^
MnZn850	0.31	-4.02	-10.30	-15.72	-20.49^b●^
MnZn1250	0.29	-5.46	-18.76	-25.31	-32.11^c▲^
25 °C 50%					
Control	0.27	23.49	12.93	4.51	15.80^a■^
MnZn44	0.44	-10.39	-14.13	-21.79	-30.37^a■^
MnZn850	0.44	-8.01	-12.82	-19.70	-25.47^a■^
MnZn1250	0.40	-6.13	-11.48	-21.02	-27.82^a■^

Different superscript letters^a−d^ (rows) and symbols^■●▲♦^ (columns) (Day 1 – Day 28) indicate significantly different values (*p* < 0.05)

The results of binary treatments (CuOx + MnZn) under different temperatures and soil moisture conditions are summarised in Table 3. Under all the different temperatures and soil moisture regimes (20 °C 30%; 20 °C 50%; 25 °C 30%; 25 °C 50%), only the CuOx500 + MnZn850 mg/kg and CuOx1000 + MnZn 1250 mg/kg treatment groups were significantly different (*p* < 0.05) from the control. However, only under 20 °C 50% environmental conditions were both these treatments (CuOx500 + MnZn850 mg/kg and CuOx 1000 + MnZn 1250 mg/kg) significantly different (*p* < 0.05) from each other.

**Table 3:** The relative growth rate (RGR) (%) of E. andrei in the binary treatment of copper oxychloride + mancozeb (CuOx + MnZn) [CuOx200 + MnZn44 mg/kg; CuOx500 + MnZn850 mg/kg; CuOx1000 + MnZn1250 mg/kg] over a 28-day period under different temperature and so

	RGR (%)				
Treatment (mg/kg)	Day 1	Day 7	Day 14	Day 21	Day 28
20 °C 30%					
Control	0.46	-3.78	-8.27	-19.41	-28.93^a■^
CuOx200 + MnZn44	0.46	-11.16	-16.98	-25.91	-31.01^a■^
CuOx500 + MnZn850	0.40	-22.15	-30.05	-35.47	-41.19^b●^
CuOx1000 + MnZn1250	0.39	-20.44	-36.14	-41.59	-50.42^b●^
20 °C 50%					
Control	0.38	5.77	0.53	-10.70	-20.06^a■^
CuOx200 + MnZn44	0.41	-1.86	-10.56	-15.97	-24.64^a■^
CuOx500 + MnZn850	0.36	-7.74	-11.31	-21.87	-27.21^b●^
CuOx1000 + MnZn1250	0.36	-18.88	-36.03	-40.02	-48.80^c▲^
25 °C 30%					
Control	0.30	23.06	16.55	10.62	1.74^a■^
CuOx200 + MnZn44	0.31	11.17	1.98	-1.58	-8.31^a■^
CuOx500 + MnZn850	0.29	3.90	-5.93	-13.13	-18.60^b●^
CuOx1000 + MnZn1250	0.30	-10.84	-18.09	-30.82	-32.06^b●^
25 °C 50%					
Control	0.27	23.49	12.93	4.51	15.80^a■^
CuOx200 + MnZn44	0.42	-13.65	-16.72	-17.94	-26.85^a■^
CuOx500 + MnZn850	0.37	-15.93	-17.98	-28.68	-37.58^b●^
CuOx1000 + MnZn1250	0.40	-8.99	-21.23	-24.82	-38.12^b●^

Different superscript letters^a−d^ (rows) and symbols^♦▲●■^ (columns) (Day 1 – Day 28) indicate significantly different values (*p* < 0.05).

#### Avoidance response behaviour

**Fig. 2:**
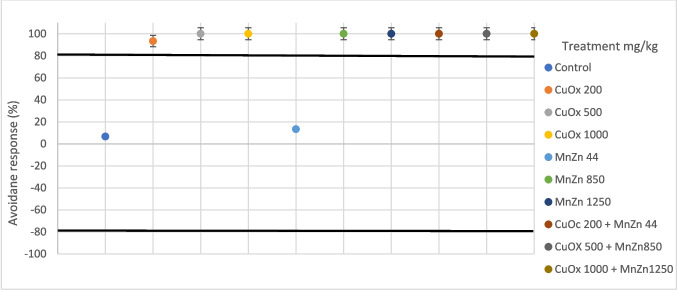
Avoidance response behaviour (%) of *E. andrei* under 20 °C 30% (temperature and soil moisture) after exposure to copper oxychloride (CuOx) and mancozeb (MnZn) in single and binary mixture

**Fig. 3:**
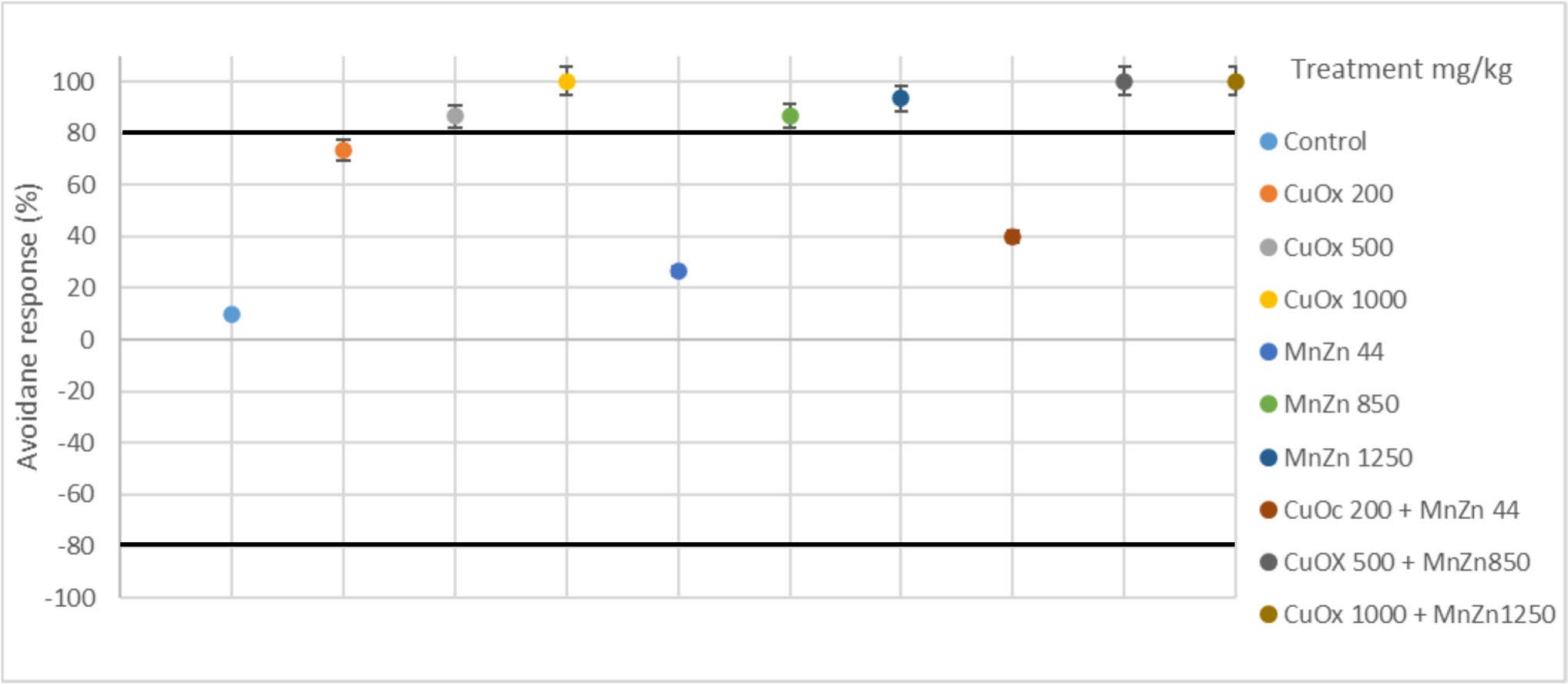
Avoidance response behaviour (%) of E. andrei under 20 °C 50% (temperature and soil moisture) after exposure to copper oxychloride (CuOx) and mancozeb (MnZn) in single and binary mixtures (*n* = 30)

There were no mortalities or cases of missing earthworms recorded during the 48-h exposure duration. The avoidance response behaviour of earthworms exposed to CuOx and MnZn in single and binary mixtures under 20 °C 30% exposure conditions is illustrated in Fig. 2. All treatments showed an avoidance response (> 80%) throughout the 48-h exposure, except for the treatments with MnZn44 mg/kg and CuOx 200 mg/kg, with a percentage avoidance response of 33.33% and 73.33%, respectively. The avoidance response behaviour under the 20 °C 50% exposure regime indicates that CuOx200 mg/kg, MnZn44 mg/kg and the binary mixture of CuOx200 + MnZn44 mg/kg had no response, with (73.33%), (26,67%) and (40%) respectively, while the rest of the treatments showed avoidance response (Fig. 3).

**Fig. 4:**
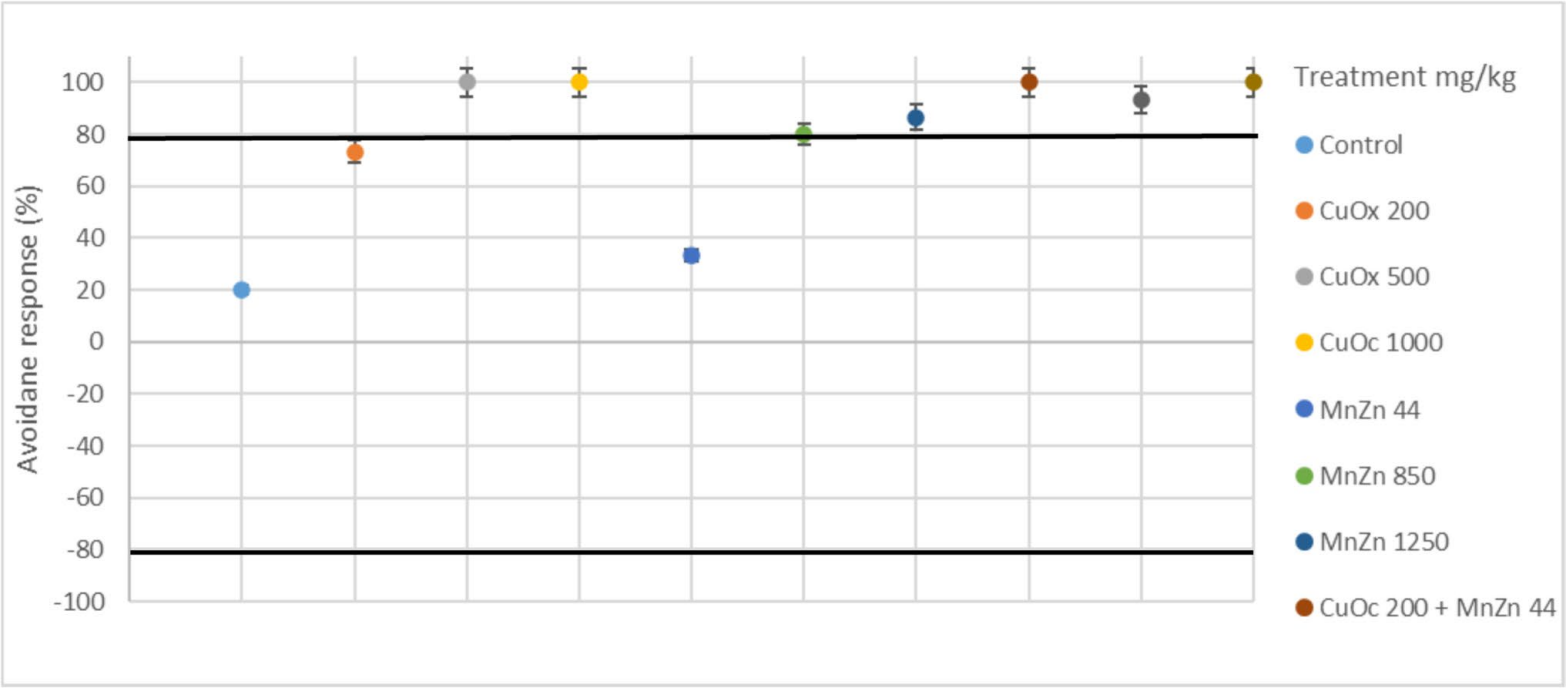
Avoidance response behaviour (%) of *E. andrei* under 25°C30% (temperature and soil moisture) after exposure to copper oxychloride (CuOx) and mancozeb (MnZn) in single and binary mixtures (*n* = 30)

**Fig. 5:**
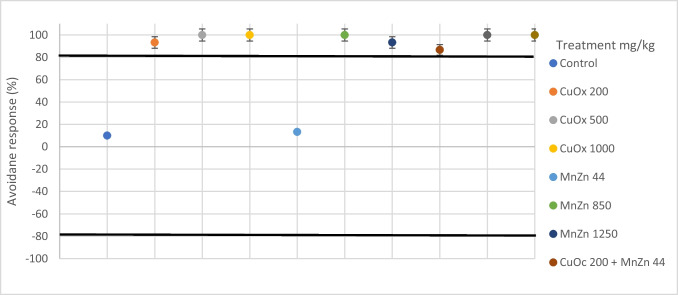
Avoidance response behaviour (%) of E. andrei under 25°C50% (temperature and soil moisture) after exposure to copper oxychloride (CuOx) and mancozeb (MnZn) in single and binary mixtures (*n* = 30)

All treatment groups in the increased temperature and reduced moisture condition (25 °C 30%) showed avoidance response behaviour, except the CuOx200mg/kg (73.33%) and the MnZn44mg/kg (33.33%) group, which indicated responses below the 80% mark (Fig. 4). However, maintaining the same temperature but with increased moisture (25 °C 50%) had all positive avoidance responses, except in the MnZn44mg/kg treatment group (13.33%) (Fig. 5).

#### Reproduction

**Fig. 6:**
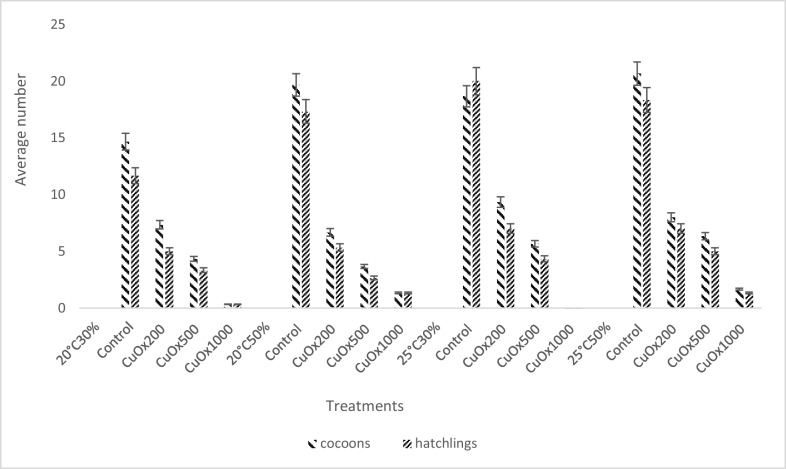
The average number of cocoons of the copper oxychloride (CuOx) treatments (CuOx200 mg/kg; CuOx500 mg/kg; CuOx1000 mg/kg) under different temperatures and moistures. (*n* = 30)

Reproductive success was determined by counting the number of cocoons and the number of juveniles hatched during the 56-day period. The average number of cocoons, the average number of hatchlings, and the number of hatchlings per cocoon are all shown in Fig. 6. The highest average (*p* < 0.05) number of cocoons and hatchlings produced in the control groups was in the 25 °C 50% treatment group, with 20.67 ± 2.52 and 18.33 ± 1.15 as the average number of cocoons and the number of hatchlings, respectively. The varying temperatures and moistures could have had an effect on the reproduction. The average number of cocoons and the number of hatchlings in the CuOx treatment decreased significantly (*p* < 0.05) as the concentration increased for all the temperature-moisture combinations. There was no significant difference (*p* > 0.05) between the control and the number of hatchlings per cocoon in all treatment groups. The lowest reproductive success was observed in the highest concentration of CuOx treatment (CuOx1000 mg/kg) in all temperature-moisture groups. However, there was no cocoon production in the CuOx1000 mg/kg treatment group under the 25°C30% treatment.

**Fig. 7:**
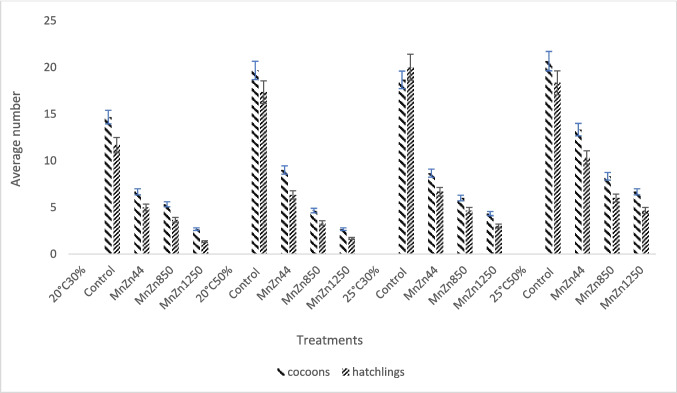
The average number of cocoons of the mancozeb (MnZn) treatment (MnZn44 mg/kg; MnZn850 mg/kg; MnZn1250 mg/kg) under different temperatures and moistures. (*n* = 30)

Similar to the results of the CuOx treatments, the reproductive output for the MnZn treatments under different temperatures and moistures increased as the concentration increased (Fig. 7). There was a significant difference (*p* < 0.05) in the number of cocoons and the number of hatchlings between the control and all the different treatment groups. The least productivity was observed in the MnZn1250 (20 °C 30%) treatment group, with an average cocoon production of 2.67 ± 0.58 and an average number of hatchlings of 1.33 ± 0.58. There was no significant difference (*p* > 0.05) between the control and the number of hatchlings per cocoon in all treatment groups.

**Fig. 8:**
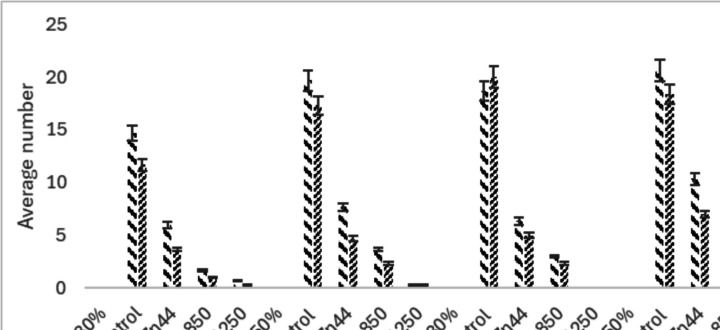
The average number of cocoons of the binary mixture copper oxychloride + mancozeb (CuOx + MnZn0 treatment (CuOx200 + MnZn44 mg/kg; CuOx500 + MnZn850 mg/kg; CuOx1000 + MnZn1

The reproductive output of the binary mixture of fungicides (CuOx + MnZn) is presented in Fig. 8. Comparatively, the results of the mixture toxicity showed trends similar to those of the exposure to a single fungicide. There was a significant difference (*p* < 0.05) in the number of cocoons and hatchlings between the control and treatment groups. Reproduction was not recorded at the highest treatment concentrations (CuOx1000 + MnZn1250 mg/kg) under temperature and moisture at 20 °C 20%. The lowest number of cocoons (0.33 ± 0.58) and hatchlings (0.33 ± 0.58) was recorded in the highest treatment concentrations (CuOx1000 + MnZn1250 mg/kg) but under the 20 °C 50% temperature-moisture condition.

## Discussion

### Mortality

Mortality was the endpoint of the earthworm acute toxicity test, but it is unlikely to be the most sensitive or ecologically relevant parameter at molecular, biochemical and physiological levels (Velki and Ečimović, 2015). Mortality did not exceed 10% (validity for the control groups) for all treatments except for CuOx1000, CuOx500 + MnZn850 and CuOx1000 + MnZn1250 mg/kg. Kilpi-Koski et al., (2020) tested the effect of binary copper, chromium and arsenic mixtures on the earthworm *E. andrei*. It was reported that 557 mg/kg of Cu did not affect the survival of *E. andrei.* Vermeulen et al. (2001) also tested the sublethal and acute toxicity of mancozeb to *E, fetida* and reported no mortality with mancozeb concentrations below 400 mg/kg. Oladipo et al. (2019) observed an increase in mortality within the artificial spiked soils of *E. fetida* when exposed to mancozeb concentrations of 800 and 1250 mg/kg, as well as copper oxychloride concentrations of 675 and 1000 mg/kg. In this study, mortality in the CuOx1000 and CuOx1000 + MnZn1250 mg/kg groups exceeded 10% in all combinations of temperature and moisture, but in the treatment of CuOx500 + MnZn850 mg/kg, significant mortality was only observed at exposure at 20 °C 30%. A study by Maboeta and Fouche (2014) on soil bioassays from a copper manufacturing site found that copper concentrations of 6925 µg g^−1^ resulted in a mortality rate of 37%. The results of this study indicated that different temperatures and soil moistures influenced the ecotoxicity of fungicides and the increased mortality of *E. andrei* exposed to single and binary mixtures of copper oxychloride and mancozeb. However, a significant increase in mortality was only observed at higher concentrations, making mortality a less sensitive endpoint than other established earthworm biomarkers. An increase in mortality will negatively impact earthworms on individual and population levels. It will reduce the reproductive output of the population as well as the ecological impact of earthworms in their environment.

### Changes in biomass of earthworms

As a result of climate change, soil temperature and soil moisture are significant factors that will affect earthworm biomass, survival, fecundity, and behaviour, and indirectly affect the soil environment and food availability (González-Alcaraz and Van Gestel 2016; Kaka et al. 2021). The relative growth rate of the earthworms under the different temperatures and soil moisture exposures varied between the different treatments. The growth rates in the copper oxychloride and mancozeb treatment groups decreased with increasing concentration. There were significant differences between the different treatments, although the most significant decrease in growth rate was observed at the highest concentrations (CuOx1000, MnZn1250 and CuOx1000 + MnZn1250 mg/kg) and 25 °C 30%. Oladipo et al. (2019) observed a significant negative impact on the biomass of earthworms when exposed to copper oxychloride at higher concentrations (675 and 1000 mg/kg). Furthermore, studies by Helling et al. 2000; Maboeta et al. 2004; Eijsackers et al. 2005 found that copper oxychloride adversely affected earthworm biomass at concentrations ranging from 519–883 µgg^−1^. Although these studies did not consider varying temperatures and soil moisture, copper oxychloride affected the relative growth rate of the earthworms.

Lima et al. (2015) tested carbaryl in LUFA 2.2 standard soil with concentrations between 20–100 mg/kg at temperatures varying between 28, 20 and 8 °C. They found that higher temperatures had more adverse effects on *E. andrei.* González-Alcaraz and Van Gestel (2016) exposed earthworms to arsenic, cadmium, and zinc under different temperatures and soil moisture conditions. They observed a loss of biomass that was more pronounced in the soils with the highest concentrations and at 25 °C 30%. Similarly, several studies have suggested the influence of temperature on pesticides (Friis et al. 2004; De Silva et al. 2009; Bandeira et al. 2020). Friis et al. (2004) reported that the copper burden in *A. caliginosa* species increased with increasing drought, suggesting that soil moisture might play a significant role in copper toxicity. De Silva et al. (2009) tested carbendazim, carbofuran, and chlorpyrifos on *E. andrei* at 20 and 26 °C, indicating that survival was more sensitive at higher temperatures. Furthermore, a study by Bandeira et al. (2020) investigating *E. andrei* responses showed that imidacloprid toxicity increases with increasing temperature. Although the studies by Friis et al. (2004); De Silva et al. (2009) and Bandeira et al. (2020) did not use metal-based fungicides, the effects of varying temperatures and moisture on pesticides are evident and must be considered.

### Avoidance behaviour

No mortalities or missing earthworms were recorded during the 48 h of exposure. Both the concentrations of copper oxychloride and mancozeb treatments (CuOx200; CuOx500; CuOx1000; MnZn850; MnZn1250) (20 °C 30% and (25 °C 50%) conditions showed avoidance response behaviour (> 80%) throughout the 48-h exposure except in the MnZn44 mg/kg treatment. On the contrary, the 20 °C 50% and 25 °C 30% exposure regimes presented an avoidance response behaviour for all treatments CuOx200 mg/kg, MnZn44 mg/kg and the binary mixture of CuOx200 + MnZn44 mg/kg. This indicates that the earthworms did not actively avoid substrates with low concentrations of mancozeb and copper oxychloride. However, varying temperatures and soil moisture impacted the avoidance behaviour of earthworms to both fungicides. Although this test is rapid, there may be different results under prolonged exposure. Jordaan et al. (2012) tested the pesticide azinphos-methyl (20–100 mg/kg) on *E. andrei* species at 20 °C and 35–40% soil moisture and found that it did not cause any avoidance in *E. andrei* species. Although this study did not have varying temperatures and soil moisture, it did indicate the relevance of pesticide avoidance behaviour tests. Oladipo et al. (2019) tested the effects of *Bacillus cereus* on the ecotoxicity of metal-based fungicide-spiked soils spiked with metal fungicides towards the earthworm species *E. andrei* at 22 °C and 60% soil moisture. They found that at lower concentrations of mancozeb (8 and 44 mg/kg), earthworms significantly preferred inoculated substrates. In comparison, at high concentrations (800 and 1250 mg/kg), earthworms completely avoided both inoculated and non-inoculated substrates. Earthworms were observed to prefer inoculated copper oxychloride substrates at 200 mg/kg, while they avoided non-inoculated substrates at the same concentration. Copper oxychloride treatments at higher concentrations (450, 675 and 1000 mg/kg) displayed a 100% net avoidance response. Although this study did not consider different temperatures or soil moisture, the results were similar, especially at higher concentrations. The fact that *Bacillus cereus* affected the avoidance response behaviour and the varying results of the CuOx200 mg/kg treatment in this study support the idea that other factors such as temperature and soil moisture changes can affect the avoidance response behaviour of earthworms. For some organisms, avoiding contaminants functions as a survival mechanism to reduce exposure to harmful substances, and the ecological consequence of their response can impact populations in the same way as lethal effects, possibly leading to population extinction (Gainer et al. 2022).

### Reproduction

The results of this study indicate that copper oxychloride and mancozeb significantly reduced the reproductive capacity of *Eisenia fetida*. Increased temperature and reduced moisture induced an increase in the toxicity of the fungicides on earthworms and caused more unfavourable conditions for earthworm reproduction. It is evident from this study that the reproduction of exposed earthworms in all treatment groups was concentration dependent and influenced by the varying temperatures and soil moisture conditions. No juveniles or cocoons were produced in the CuOx1000 mg/kg treatment at (25 °C 30%), indicating that copper oxychloride may be more toxic than mancozeb, especially under drought conditions (increased temperature and reduced moisture). Oladipo et al. (2019) reported a similar trend in which neither cocoons nor juveniles were observed at 675 and 1000 mg/kg of copper oxychloride exposure. Maboeta and Fouche (2014) also reported that cocoon production was severely reduced in field soils with copper concentrations up to 1400 mg/kg. Although Oladipo et al. (2019) used standard temperature and soil moisture (20 °C and 60%), while Maboeta and Fouche (2014) used 25 °C and 60%, the effects in both experiments were similar. However, the findings of this study indicate that changes in temperature and soil moisture may have altered the bioavailability of copper oxychloride and mancozeb to earthworms and thus their reproductive output. This study found a significant variation in the number of hatchlings in the CuOx200 mg/kg treatment between 20 °C and 25 °C when comparing the same treatments under different temperatures and soil moistures. Similarly, in the CuOx500 mg/kg group, there was a significant difference between 20 °C and 25 °C in the number of cocoons produced. Although this trend was not observed in the CuOx1000 mg/kg treatment, it indicates a significant response at different temperatures. The 20°C50% exposure had the lowest average number of hatchlings and cocoons for the CuOx200 and CuOx500 mg/kg treatment. The CuOx1000 mg/kg and 25 °C 30% exposure had the lowest average number of hatchlings and cocoons. Under the highest concentration, high temperature and low soil moisture had the most detrimental effect on earthworm reproduction. Although this study observed a lower mean number of cocoons and hatchlings under MnZn850 and MnZn1250 mg/kg treatments, Oladipo et al. (2019) (22 °C and 60% moisture) reported no cocoons or juveniles with MnZn800 and MnZn1250 mg/kg. The disparity in the number of cocoons may be due to the different moisture and temperature conditions. Dry soils can result in cocoon dehydration, which may prevent embryonic development (Lowe and Butt 2005). The implications of climate change (increasing temperature and decreasing soil moisture) will negatively impact the fecundity of earthworm populations. Vermeulen et al. (2001) reported no statistically significant differences between the control and the MnZn8 and MnZn44 mg/kg treatments (25 °C and 75% moisture). This report looked similar to the results obtained in this study under the 25 °C 30% condition. However, comparing the same treatments under different temperatures and soil moisture levels revealed a significant difference in the number of cocoons and hatchlings. This finding implies that soil moisture and temperature played a significant role in the reproductive success of earthworms. Significant differences (*p* < 0.05) in the number of cocoons, as well as the number of hatchlings between the control groups and the binary treatments (CuOx200 + MnZn44 mg/kg, CuOx500 + MnZn850 mg/kg and CuOx1000 + MnZn1250 mg/kg) under the different temperature-moisture conditions. In the 25 °C 30% exposure, there was only a significant difference (*p* < 0.05) in the number of cocoons at the highest concentration (CuOx1000 + MnZn1250 mg/kg). The above result is also consistent with the findings of the Mancozeb experiment. Furthermore, at 25 °C 30%, the CuOx1000 + MnZn1250 mg/kg treatment no juveniles or cocoons were produced, and this was also observed in the CuOx1000 mg/kg treatment under the same temperature-moisture conditions. It suggests that the single CuOx1000 mg/kg exposure had the same outcome as the binary treatment with CuOx1000 + MnZn1250 mg/kg. Thus, the presence of mancozeb had no synergistic effect on the copper oxychloride. This study also demonstrated that, while temperature and soil moisture influenced the ecotoxicity of copper oxychloride and mancozeb, higher temperatures with lower moisture content had the most adverse effects on reproduction. Most earthworm tissues are assumed to be vulnerable to copper because they cannot synthesise copper-binding ligands in response to the metal (Helling et al. 2000).

## Conclusions

Copper oxychloride was responsible for a significant portion of the reported mortality of earthworms in the treatment groups, except for the CuOx200 and CuOx500 groups, which recorded a mortality of less than 10% in all treatment regimes. Mancozeb was not as hazardous as copper oxychloride. The mixture treatment, CuOx1000 + MnZn1250, has the highest significant mortality rate. Copper oxychloride and mancozeb did not show any form of synergistic or antagonistic toxicity under the different climatic variables. Increased concentrations of both contaminants were associated with decreased biomass and reproduction and increased avoidance behaviour. Temperature and moisture were significant factors in earthworm mortality, biomass changes, avoidance behaviour and reproduction. As a result, climate change is likely to significantly impact the outcomes of metal ecotoxicity to earthworms and their ecological activities. However, for a more detailed understanding of soil metal pollution under current global climate change scenarios, we suggest a combination of more environmental variables and broader-scale field investigations.

## Data Availability

The authors declare that the data supporting the findings of this study are available within the paper. Should any raw data files be needed in another format they are available from the corresponding author upon reasonable request.
